# Chitosan nerve conduits seeded with autologous bone marrow mononuclear cells for 30 mm goat peroneal nerve defect

**DOI:** 10.1038/srep44002

**Published:** 2017-03-13

**Authors:** Aikeremujiang Muheremu, Lin Chen, Xiyuan Wang, Yujun Wei, Kai Gong, Qiang Ao

**Affiliations:** 1 Department of tissue engineering, School of fundamental sciences, China Medical University, Shenyang, Liaoning Province, 110122, China; 2 Department of spine surgery, Sixth Affiliated Hospital of Xinjiang Medical University, Urumqi, Xinjiang, China; 3 Department of Neurosurgery, Tsinghua University Yuquan Hospital, Beijing, 100049, China; 4 Department of tissue engineering, China Medical University, Shenyang, Liaoning Province, 110122, China

## Abstract

In the current research, to find if the combination of chitosan nerve conduits seeded with autologous bone marrow mononuclear cells (BM-MNCs) can be used to bridge 30 mm long peroneal nerve defects in goats, 15 animals were separated into BM-MNC group (n = 5), vehicle group (n = 5), and autologous nerve graft group (n = 5). 12 months after the surgery, animals were evaluated by behavioral observation, magnetic resonance imaging tests, histomorphological and electrophysiological analysis. Results revealed that animals in BM-MNC group and autologous nerve graft group achieved fine functional recovery; magnetic resonance imaging tests and histomorphometry analysis showed that the nerve defect was bridged by myelinated nerve axons in those animals. No significant difference was found between the two groups concerning myelinated axon density, axon diameter, myelin sheath thickness and peroneal nerve action potential. Animals in vehicle group failed to achieve significant functional recovery. The results indicated that chitosan nerve conduits seeded with autologous bone marrow mononuclear cells have strong potential in bridging long peripheral nerve defects and could be applied in future clinical trials.

Although the peripheral nervous system has the ability to regenerate after injury, functional recovery after the injury is still unsatisfactory. For repairing long peripheral nerve defects, autologous nerve grafting is still the gold standard. However, this technique has several disadvantages such as limited graft supply, donor site morbidity and prolonged operation time. With the application of nerve conduits and cell transplantation technique, surgeons now have various options for treating peripheral nerve defects. Chitosan nerve conduits have been reported to be effective in bridging peripheral nerves^1^. However, because of little cellular and neurotropic support in hollow nerve conduits, they can only be used to bridge short nerve defects. Seeding conduits with appropriate cells have been described as a possible approach to solve this problem^2^. They were found to be scattered inside the tubes in a manner similar to that of bands of Bunger, and secrete neurotrophic factors that can create microenvironment which significantly accelerates axonal regeneration^3^,^4^. For example, Fansaet *et al*.^5^ cultured isogenic Schwann cells (SCs) and implanted them into several types of acellular autologous matrixes including veins, muscles, nerves, and epineurium tubes. The muscle–SC graft showed a systematic and organized regeneration, including a proper orientation of regenerated fibers.

Although Schwann cells are the main supporting cells for peripheral nerve regeneration, harvesting enough Schwann cells needed for treatment is impossible in most clinical settings. On the other hand, bone marrow mononuclear cells (BM-MNC) are readily available and can be easily harvested. If those cells can be proved to be effective in repairing long peripheral nerve defects in large animal models, they would be available for further tests in clinical trials^6^. Unfortunately, most of the current literature is about the allogeneic cell transplantation which inevitably leads to poor functional recovery due to immunological rejection^7^,^8^,^9^. The current study was carried out to test the curative effect of chitosan tubes seeded with autologous BM-MNCs in repairing 30 mm goat common peroneal nerve defect.

## Methods

### Preparation of chitosan tubes

**Figure 1:**
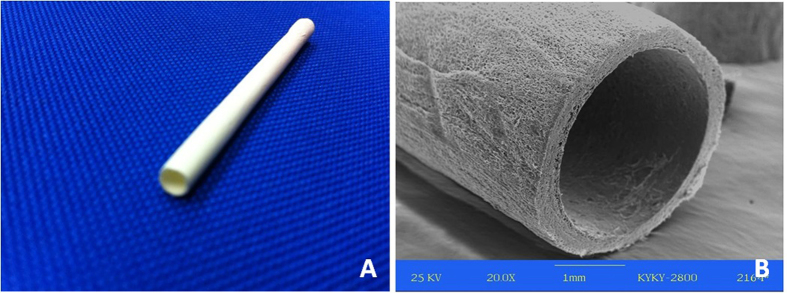
The chitosan nerve conduits were manufactured with freeze drying method. Inner diameter of the conduit was 3 mm and the wall of the conduit was 0.5 mm thick. They were sterilized in 75% ethanol for 2 hours and washed in sterilized water for 10 minutes before use.

The chitosan nerve conduits were manufactured with freeze drying method, which was previously described by our team^7^,^8^. Inner diameter of the conduit was 3 mm and the wall of the conduit was 0.5 mm thick (Fig. 1). Nerve conduits were sterilized in 75% ethanol for 2 hours and washed in sterilized water for 10 minutes before use.

### Preparation of BM-MNCs

**Figure 2:**
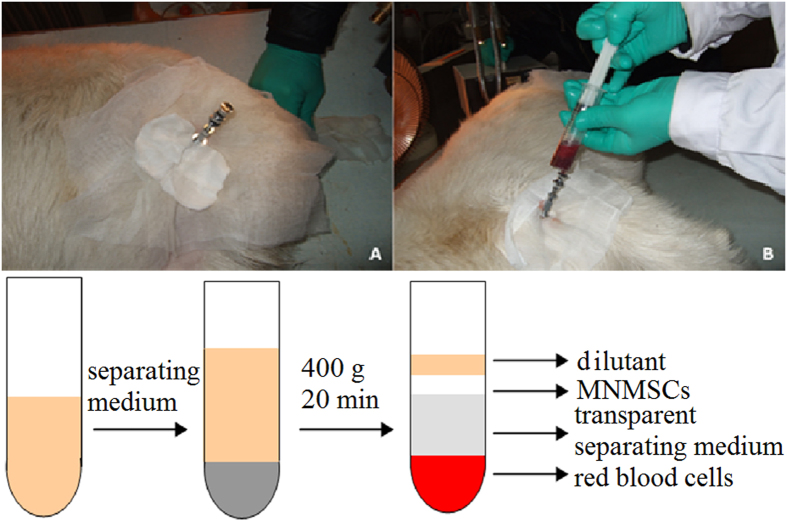
The mononuclear cells in bone marrow were extracted by gradient centrifugation method.

**Figure 3:**
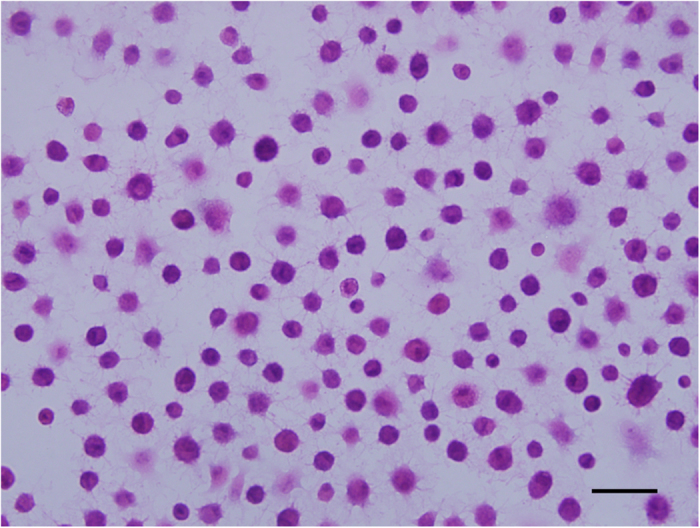
Mononuclear cells in bone marrow after extraction.

All the animal experiments in the current research were carried out in accordance with the US National Institute of Health Guide for the Care and Use of Laboratory Animals (NIH Publications No. 80-23) revised in 1996 and approved by the Tsinghua University Animal Ethics Committee and the Beijing Administration Committee of Experiment Animals. BM-MNCs were obtained from the goats before the surgery. Bone marrow was taken from the goat femoral bones and BM-MNCs were isolated from the bone marrow by density gradient centrifugation (Fig. 2), then maintained in L-DMEM containing 15% fetal bovine serum (FBS) at 37 °C in a humidified atmosphere with 5% CO_2_. BM-MNCs with the cell density of 1 × 10^8^/0.5 ml were applied for cell transplantation (Fig. 3).

### Animals

15 healthy goats (male or female, weighted 20–25 kg) provided by Beijing Experimental Animal Center were divided into 3 groups with each group including 5 animals. BM-MNC group: chitosan nerve tube +1 × 10^8^ autologous BM-MNCs in 0.5 ml L-DMEM; Vehicle group: chitosan nerve tubes with 0.5 ml L-DMEM; Nerve graft group: autologous nerve transplant.

### Ethical approval

All the animal experiments were approved by the ethical committee of Tsinghua University. The procedures were accordance with the ethical standards of Helsinki Declaration.

### Surgical procedure

**Figure 4:**
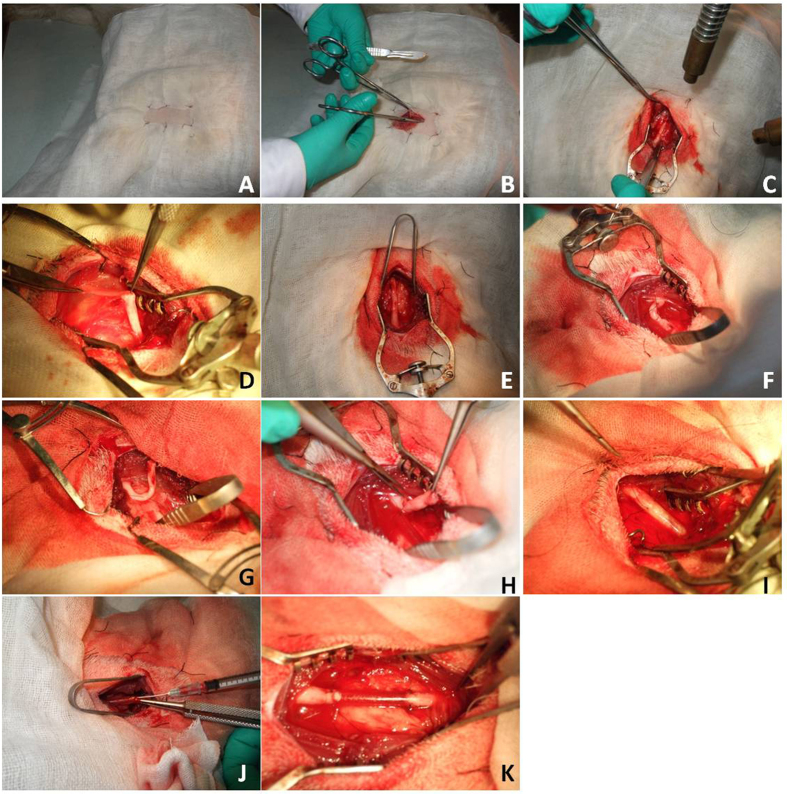
After preparation and disinfection of the skin (**A**), a left postero-lateral femoral incision was made (**B**) and the general peroneal nerve was exposed (**C**–**G**). 30 mm general peroneal nerve was removed 1 cm distal to its starting point (**H**) and the defect was bridged by a 34 mm chitosan nerve conduit, peroneal nerve was inserted 2 mm into the conduit at each side. Epineurium of the peroneal nerves were sutured to the nerve conduits by four stiches of 8–0 nylon sutures at each side (**I**). 1 × 108/0.5 ml BM-MNCs was injected into the conduit after the bridging was completed (**J**,**K**).

Bridging the peroneal nerve defect with chitosan nerve conduits seeded with autologous BM-MNCs (BM-MNC group): the goats were anesthetized by intravenous administration of 1% pentobarbital sodium (Shanghai Xianfeng Co. Ltd., Shanghai, China) at a dose of 40 mg/kg. Sciatic nerve and its branches were exposed by a left postero-lateral femoral incision. 10 mm length segment of general peroneal nerve was removed from 10 mm distal to the bifurcation point of sciatic nerve and the defect was bridged by a 34 mm chitosan nerve conduit, and the peroneal nerve was inserted 2 mm into the conduit at each side. Epineurium of the peroneal nerves was sutured to the nerve conduits by four stiches of 8–0 nylon sutures. 0.5 ml L-DMEM containing 1 × 10^8^ BM-MNCs was injected into the conduit after the bridging was completed (Fig. 4).

#### Chitosan conduit bridging (Vehicle group)

All the procedures were the same as the BM-MNC group, but instead of autologous BM-MNCs, 0.5 ml L-DMEM was injected into the conduit after bridging the peroneal nerve.

#### Autologous nerve grafting

The operational steps are similar to the chitosan conduit bridging, except that the 30 mm defect created on the peroneal nerve was bridged by grafting the removed nerve itself using 8–0 nylon sutures.

### Behavioral analysis

Skin and muscles of the limb at the side of surgery and the walking patterns of the animals were analyzed once each day after the surgery until the animals were sacrificed 12 months after the surgery.

### MRI tests

12 months after the surgery, all the animals were anesthetized and the peroneal nerves were scanned with high resolution MRI scanner (Philips Achieva 3.0T TX) 3D multi-echo fast Spin-Echo with water selective sequence (3D mFFE WATs). Image parameters: field of view (FOV): 271 × 171 mm, matrix: 456 × 283, TR 23 ms, TE 9.2/5.1 ms, time of scan: 6 min 32 sec. Three-Dimensional Video Postproduction include maximal intensity projection (MIP) and multi planar reconstruction (MPR).

### Electrophysiological tests

After the MRI test, proximal and distal ends of the grafted nerve or the conduit were exposed, an electro-physiological test instrument (RM6240 biomedical signal acquisition and processing system, Chengdu instrument factory, Sichuan, China) was used to test the compound muscle action potentials (CMAPs). Stimulating electrode was placed on the peroneal nerve at proximal end of the suture, and the recording electrode was pierced into the anterior tibial muscle and the conduction velocity of both left and right peroneal nerves was measured.

### Histomorphometry

After the electrophysiological studies, animals were euthanized under deep anesthesia. The peroneal nerves were taken from the middle and the distal end of the nerve conduit or autograft, fixed in 4% paraformaldehyde in 4 °C for 48 hours and cryoprotected in 20% sucrose for 24 hours; nerves were cut into 10 μm slides in −20 °C cryostat (CM1900, Leica, Germany); Slides were blocked by 5% goat serum for an hour. The primary antibodies: rabbit anti-neurofilament protein antibody (1:200, Sigma) and mouse anti-S100 protein antibody (1:500, Sigma) were added on the slides, and the slides were incubated at 4 °C for 24 hours. Then the slides were washed with PBS 3 times, 5 minutes each time, incubated with secondary antibody (fluorescein isothiocyanate (FITC) labeled goat anti-rabbit IgG antibody (ZSGB-biotechnologies, Beijing, China) and rhodamine labeled goat anti-mouse antibody (ZSGB-biotechnologies, Beijing, China) at room temperature for 2 hours and mounted after further PBS washing. The slides were observed using confocal microscope (FV10i, Olympus, Japan) after immunohistochemical staining.

### Toluidine blue staining of the semi-thin sections

Semi-thin sections were prepared from nerve segments with the transection area of 1 mm^2^ and length of 2–3 mm. Those segments were taken 1 cm from the proximal end and the distal end of the nerve conduit or autograft. After being washed in phosphate buffer, the nerve segments were osmicated in osmic aced fixation buffer (1% osmium tetroxide, 0.1 M phosphate buffer) at 4 °C for an hour. Then the nerve segments were washed for several times, dehydrated with ethanol, and embedded in Epon812. 1 μm semi-thin sections were cut by ultramicrotome, stained with toluidine blue and observed using light microscope (BX41, Olympus, Japan). Morphometric parameters included the myelinated axon density, myelin sheath thickness, axon diameter in three randomized fields of interest.

### Image capturing and analysis

The slides were observed under light microscope (BX41, Olympus, Japan). 3 visual fields with more than 1000 nerve fibers were chosen and magnified by 600 times before pictures of semi thin sections were captured to quantitatively evaluate the capacity of different methods for neural regeneration. Image-Pro Plus 5.0 (Media Cybernetics, USA) was used to measure the density of the nerve fibers, diameter of the axons and the thickness of the myelin sheath.

### Statistical analysis

Independent sample t-tests, paired sample t-tests and X^2^ analysis were performed using SPSS17.0 (Philadelphia, USA) analytical software to compare the curative effect of different treatment options, and difference was considered significant when P < 0.05.

## Results

### Behavioral analysis

Typical symptoms of the goat with injured peroneal nerve include foot drop sign, difficulty in plantar flexion, difficulty in lifting up the foot and toes and making toe-out movements, damage or ulcer of the skin in the surgery side and dystrophy of the anterior tibial muscle. After the surgery, all the animals demonstrated difficulty in movements of the injured limb. During the 12 months after surgery, animals from BM-MNC group and nerve graft group showed gradual improvement in the function of injured limb; animals in vehicle group failed to achieve significant functional recovery and suffered from muscle dystrophy and skin ulcer at the injury side.

**Figure 5:**
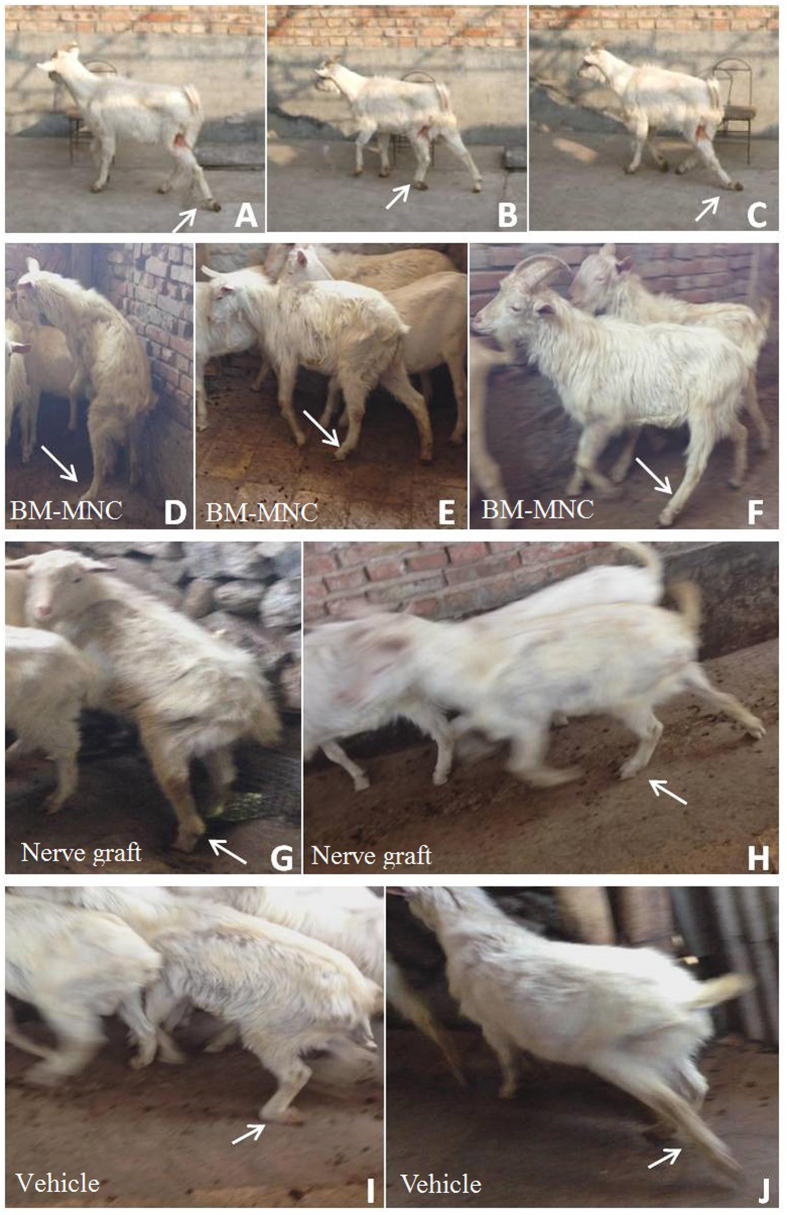
(**A**–**C**) One week after the surgery, the animals of any group had drop-foot and plantar flextion posture. The foot was dragged when walking (arrow). (**D**–**F**) Animals in BM-MNC group 12 months after the surgery can stand by their hind feet and freely move the foot at the surgery side (arrow) (**G**,**H**) Animals in nerve graft group12 months after the surgery are similar to the animals in BM-MNC group (arrow) (**I**,**J**) Animals in vehicle group showed foot drop sign their hind foot cannot be extended voluntarily while walking.

All the animals were alive 12 months after the surgery. No skin ulcer or obvious dystrophy of anterior tibial muscle was detected in animals from BM-MNC group and nerve graft group. They can stand on their hindlimbs, walk and run freely. Animals in vehicle group had significant dystrophy of right anterior tibial muscles; ulcer or scarring can be seen on the skin of the right foot; the right foot was flexed and cannot be extended voluntarily (Fig. 5).

### Magnetic resonance imaging

**Figure 6:**
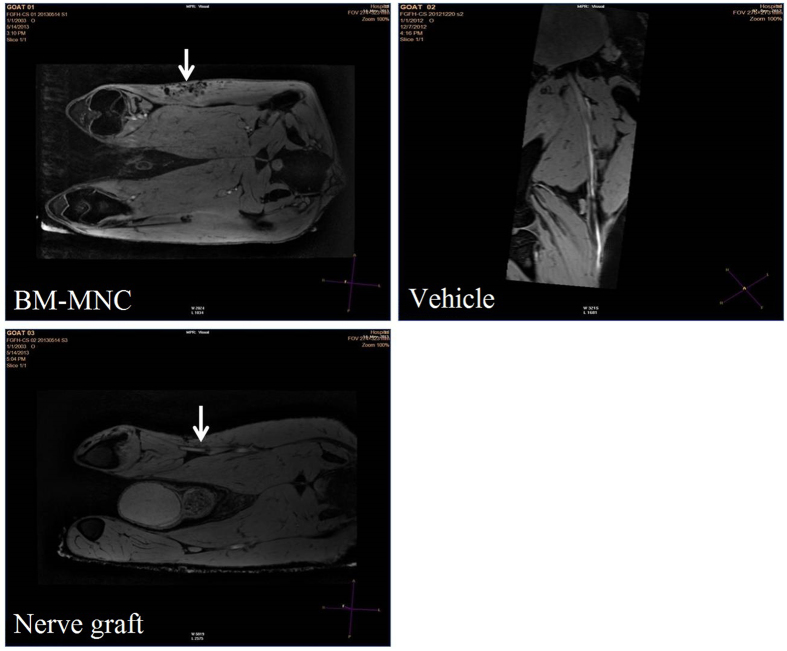
Three-dimensional reconstruction using 3D mFFE WATs demonstrated clearly the nerves, blood vessels and muscles and nerve conduits at the region of interest. In the animals from BM-MNC group, structure of the regenerated nerve was continuous with the host nerve, but the signal intensity is weaker than the normal nerves, the diameter of the grafted nerve is smaller. In the animals from vehicle group, the regenerated nerve failed to bridge the peroneal nerve defect. In the animals from nerve graft group autologous nerve grafting group), structure of the nerve graft was continuous with the host nerve, but the signal intensity is weaker than the normal nerves, the diameter of the grafted nerve is smaller than the normal side.

Anatomical abnormalities on the region of surgery were observed by magnetic resonance imaging. Three-dimensional reconstruction using 3D mFFE WATs demonstrated the nerves, blood vessels and muscles and nerve conduits at the region of interest clearly. In the animals from BM-MNC group (Fig. 6A), structure of the regenerated nerve was continuous with the host nerve, but the signal intensity is weaker than the normal nerves, the diameter of the grafted nerve is smaller. In the animals from vehicle group (Fig. 6B), the regenerated nerve failed to bridge the peroneal nerve defect. In the animals from nerve graft group (Fig. 6C), structure of the nerve graft was continuous with the host nerve, but the signal intensity was weaker than the normal nerves, the diameter of the grafted nerve was smaller than the normal side.

### Electrophysiologic tests

**Figure 7:**
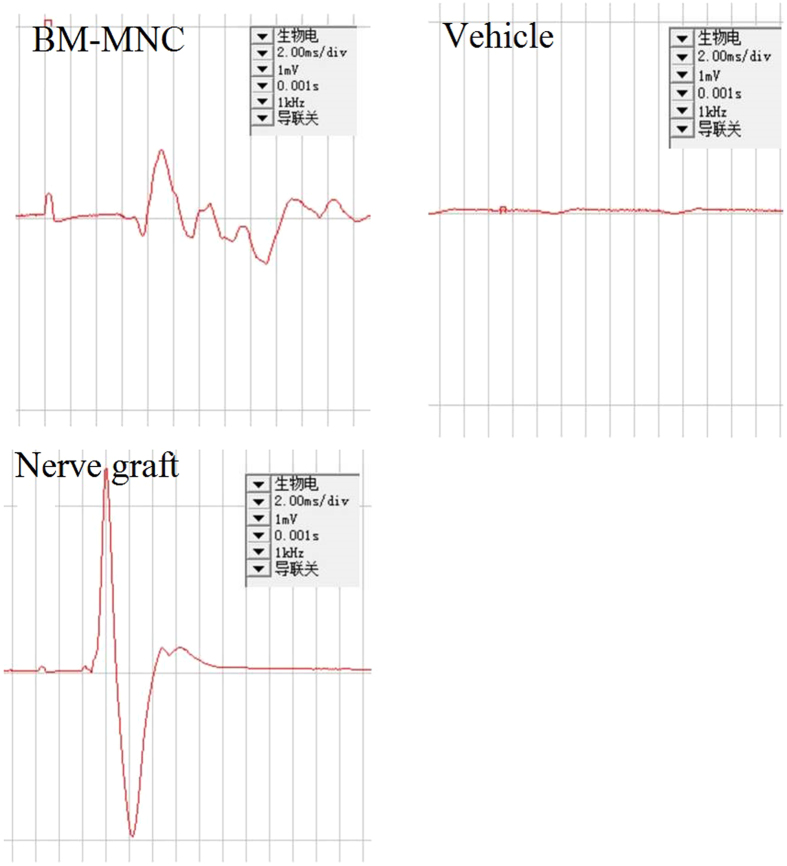
No significant difference was found comparing the conduction velocity between BM-MNC group and nerve graft group (independent sample t-tests, P > 0.05), however, the conduction velocity was lower in the left peroneal nerve comparing to the right side in both groups. In the animals of vehicle group, no action potential can be detected at the surgery side.

No significant difference was found comparing the conduction velocity between BM-MNC group and nerve graft group (independent sample t-tests, P > 0.05), however, the conduction velocity was lower in the left peroneal nerve comparing to the right side in both groups (independent sample t-tests, P < 0.05). In the animals of vehicle group, no action potential can be detected at the surgery side (Table 1, Fig. 7).

### Semi-thin slides

**Figure 8:**
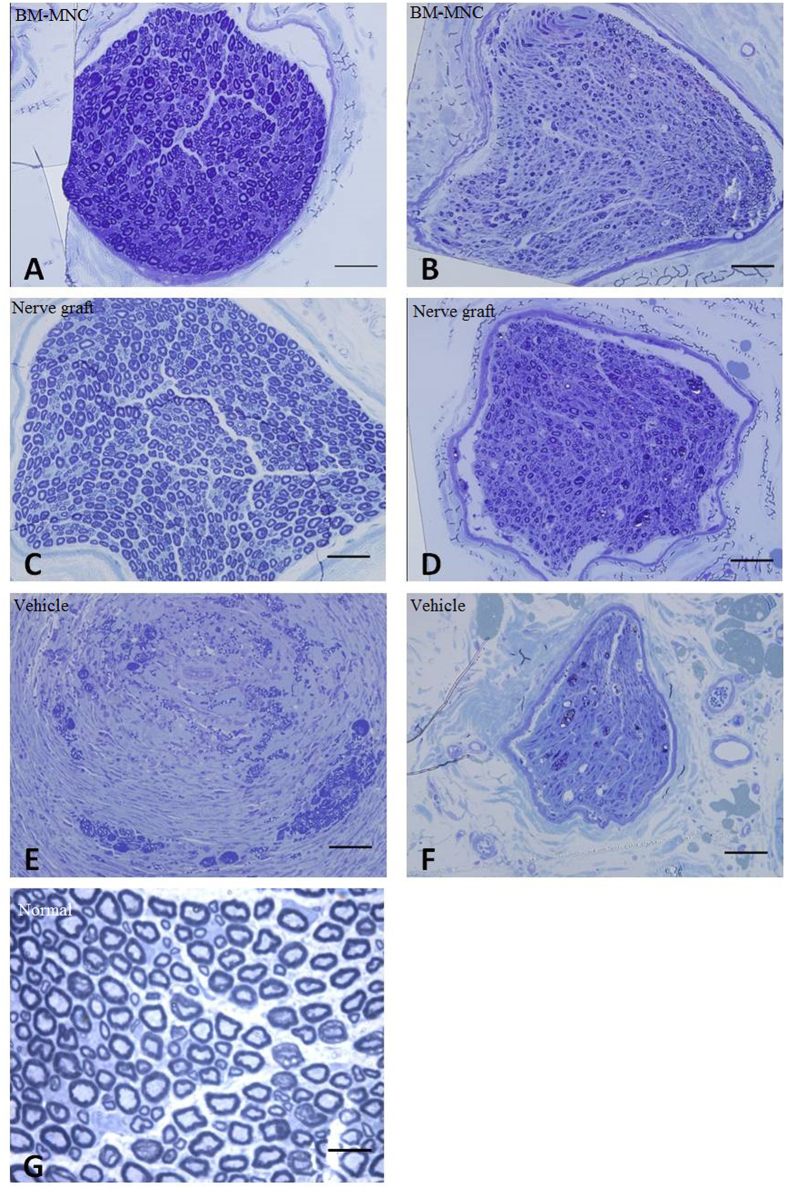
In BM-MNC group and nerve graft group, there were myelinated nerve fibers similar to normal nerve, but their diameter was significantly smaller than the normal nerves. No myelinated nerve fibers were observed in regenerated nerves in vehicle group. (**A**,**B**) BM-MNC group, regenerated axons were unevenly distributed within the nerve. (**C**,**D**) Nerve graft group, regenerated axons were evenly distributed across the cross section of the nerve. (**E**,**F**) Vehicle group, few regenerated axons can be observed at the cross section of the nerve. (**A**,**C**,**E**) Central segment of regenerated nerve. (**B**,**D**,**F**) Distal part of the regenerated nerve. (**G**) Normal peroneal nerve. Scale: 50 μm.

The central and distal portions of the regenerated nerve were stained with tubulin blue and observed under light microscope. Regenerated nerves have successfully bridged the nerve defect in all the animals in BM-MNC group and nerve graft group, but none of the animals in vehicle group. X^2^ analysis showed significant differences among groups concerning the capacity of different methods to bridge the long peroneal nerve defect (P < 0.05). The regenerated axons in BM-MNC group and nerve graft group were encapsulated with fibrous epineurium. Nerve fibers of regenerated peroneal nerves in BM-MNC group and nerve graft group have higher density, smaller axon diameter and thinner myelin sheath than the normal peroneal nerves (Fig. 8). Independent sample t-tests revealed no significant differences regarding axonal density, diameter and myelin sheath thickness between BM-MNC group and nerve graft group (P > 0.05). In animals of BM-MNC group and nerve graft group, axonal density at the injury side (right foot) was not significantly different than the intact peroneal nerves of the left foot (Paired sample T-tests, P > 0.05), but the axon diameter and myelin sheath thickness at the injury side were significantly smaller than the intact peroneal nerves of the left foot (Paired sample T-tests, P < 0.05) (Table 2).

### Immunohistochemical staining

**Figure 9:**
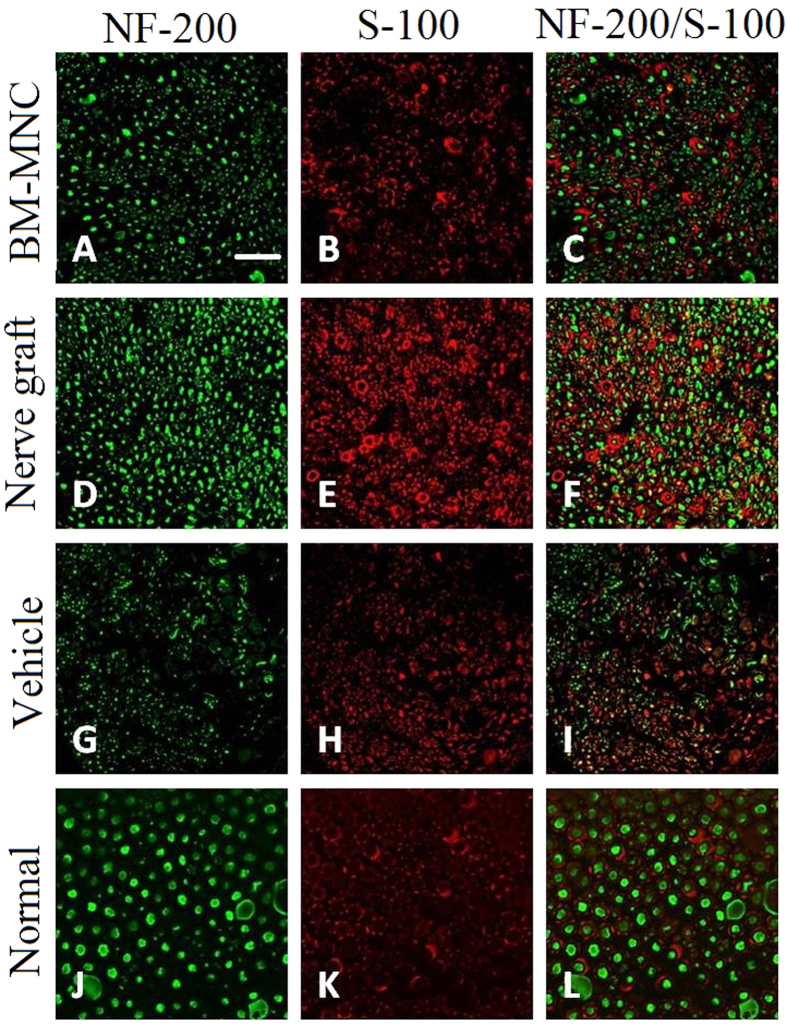
The regenerated axons in BM-MNC group and nerve graft group were encapsulated with fibrous epineurium. Nerve fibers of regenerated peroneal nerves in BM-MNC group and nerve graft group have higher density, smaller axon diameter and thinner myelin sheath than the normal peroneal nerves. (**A**–**C**) BM-MNC group; (**D**–**F**) nerve graft group; (**G**–**I**) vehicle group; (**J**–**L**) Normal nerve. Green fluorescent light is emitted by NF-200 protein labeled by FITC and shows the axons. Green fluorescent light is emitted by S-100 labeled by TRITC and shows myelin sheath.

Neurofilament protein NF-200 can be used to label the regenerated axons, FITC florescent antigen can show the regenerating axons. S-100 is a protein that can label the myelin sheath, after fluorescent labeling with second antigen, the red TRITC florescent can show the Schwann cells in the myelin sheath. In BM-MNC group and nerve graft group, there were myelinated nerve fibers similar to normal nerves. No myelinated nerve fibers were observed in regenerated nerves in vehicle group (Fig. 9).

## Discussion

Chitosan conduits used in this research were manufactured by our team by dry-freezing method, which was described in our previous publications^10^,^11^. Chitosan is a degradable natural material with high biocompatibility and degradability. We have found in a previous study^12^ that chitosan/silk fibronin based tissue engineered graft support the differentiation of human adipose-derived stem cells (ASCs) into neural stem cells, and adipose derived stem cells seeded in chitosan/silk fibronin based tissue engineered graft can significantly improve axonal regeneration and functional recovery when they were used to bridge rat sciatic nerve injury.

When silicon nerve conduit was coated with chitosan and was used to bridge rat sciatic nerve, level of interleukin-1β and leukotriene B4 in the conduit was significantly decreased and the formation of glial scarring was inhibited^13^. Xie *et al*.^14^ reported that composite material of chitosan and polylactic acid (PLA) have increased strength, elasticity and biocompatibility and can be applied to bridge 10 mm rat sciatic nerve defect with satisfying results. Gu *et al*.^15^ published a clinical case reporting the bridging of 30 mm defect of the median nerve by nerve conduit constructed with the composite material of chitosan and polyglycolic acid (PGA). After 36 months’ follow up, the patient reported satisfactory functional recovery of his fingers.

Although a combination of chitosan nerve conduits and certain other materials might result in compounds with unique abilities, since the purpose of the current research was to test the potential of clinical translation of this material, we have applied chitosan conduits only in this study. It normally takes the chitosan nerve conduits used in this research 15 months to gradually degrade in the body. Hu *et al*.^16^ used rhesus monkeys as experimental animals to use the combination of chitosan/PLGA nerve conduits and MSCs to bridge 50 mm peroneal nerve defect. The method was proved to be effective when evaluated 12 months after the surgery.

Hollow nerve conduits are limited in their ability to support axonal regeneration due to the lack of neurotropic support. Seeding them with stem cells is a viable choice to solve this problem. Nerve conduits seeded with stem cells have been extensively studied and recognized as a potential substitution for autologous nerve grafts. Authors have tried various combinations of different nerve conduits and supporting cells to repair peripheral nerve injury. Pang *et al*.^17^ reported that acellular nerve allografts seeded with BM-MNC can be more effective in repairing peripheral nerves than acellular nerve allografts alone. Nijhuis *et al*.^18^ reported that vein allografts stuffed with muscle tissues and BM-MNCs were effective in bridging 15 mm rat sciatic nerve. Hu *et al*.^16^ used Chitosan/PLGA nerve conduits seeded with MSCs to repair 50 mm median nerve defect in Rhesus monkeys and achieved certain functional recovery with no adverse side effects in the 12 months’ follow up.

Autologous BM-MNCs can be acquired in great amount with minimal invasive measures. Application of autologous BM-MNCs is not subjected to infection, immunologic rejection, tumor formation or ethical problems. BM-MNCs have been used to treat peripheral nerve injury by several studies. Goel *et al*.^19^ used BM-MNCs to treat rat sciatic nerve injury, which significantly promoted the axonal regeneration and myelin formation. Sakar *et al*.^20^ reported that the combined use of poly (3-hydroxybutyrate-co-3-hydroxyhexanoate (PHBHHx)) and human bone marrow mononuclear cell effectively promoted axonal regeneration in 1 mm rat sciatic nerve defect models. Ishikawa *et al*.^21^ used a combination of Schwann cells differentiated from BM-MNCs and chitosan nerve conduits to repair 8 mm rat peripheral nerve defects. 7 days after the surgery, migrated Schwann cells were found around the regenerating axons; one month after the surgery, myelin sheath was formed by Schwann cell like cells around the regenerating axons. Hsu *et al*.^22^ seeded BM-MNCs into the chitosan conduits imbedded with laminin and used it to bridge 10 mm rat sciatic nerve defects. 16 weeks after the surgery, functional analysis, weight of the gastrocnemius muscle and retrograde labeling tests showed that nerve conduits seeded with BM-MNCs were effective in treating peripheral nerve injury. In the study of Zheng *et al*.^23^,^24^, chitosan nerve conduits seeded with BM-MNCs were used to bridge 8 mm rat sciatic nerve defects. 6 weeks after the surgery, sciatic nerve function index, density and the diameter of the regenerated nerve fibers were not significantly different from autologous nerve grafting group, indicating that chitosan nerve conduits seeded with BM-MNCs could be a combination with great potential to repair peripheral nerve injury. However, no study was carried out to test the efficacy of chitosan nerve conduits seeded with BM-MNCs in the treatment of long nerve defects in large animal models. The current study was designed and carried out to fill this void.

Goat is an easily available and mellow natured large animal that can mimic clinical situations. Goat was chosen in the current research, and a 30 mm nerve defect was created according to the length tested in goats in the current literature^23^,^24^. Since the main purpose of this research was to find if the combined application of chitosan nerve tubes and mononuclear stem cells can have ideal long term effect in bridging long peroneal nerves. Except from the behavioral observation, tests were not carried out to evaluate the treatment effect at intermediate time points because of the high expense of large animal models. Moreover, the main role of implanted cells is providing neurotrophic support for the regenerating axons, not replacing the existing cells. In our previous research on rats, implanted cells cannot be found 4 weeks after transplantation, and we assume similar results would be achieved by the large animal models.

Considering the ethical and technical aspects of applying novel biomaterials in clinical settings, in the current study, we sought to use the chitosan nerve conduits seeded with autologous BM-MNCs to bridge long nerve defects in large mammals, to provide evidence for the clinical application of this technique. Results of the current study indicated that this combination can significantly facilitate axonal regeneration and functional recovery in goats after bridging long nerve defects.

How BM-MNCs promote axonal regeneration is not yet clear. Although we did not study its working mechanism in the current research, previous publications may provide some insight into this matter. It was reported by previous studies that BM-MNCs can be differentiated into Schwann cell like cells and produce various growth factors, creating favorable microenvironment for axonal regeneration^25^,^26^,^27^. BM-MNCs were also reported to inhibit apoptosis of the neural cells and inflammation caused by implanting chitosan nerve conduits^28^. Further research is necessary to clarify the problem.

It is possible to genetically modify the BM-MNCs to have more desirable characteristics^29^. Xin *et al*.^30^ reported chitosan conduits seeded with Schwann cells transfected with the glial cell derived neurotropic factor (GDNF) genes to be as effective as autologous nerve grafts in bridging 8 mm defects of rat sciatic nerve. However, because of the ethical considerations for applying genetically engineered cells in clinical practice, in the current study, we applied autologous BM-MNCs for cellular support with no further modification.

According to the behavioral analysis, histological observations and electrophysiological tests of this study, chitosan nerve conduits seeded with autologous BM-MNCs have the similar effectiveness to autologous nerve grafts in repairing 30 mm long peripheral nerve defects. On the other hand, chitosan conduits with no cellular support failed to bridge the nerve defect. Results of the current study indicate that chitosan nerve conduits seeded with autologous BM-MNCs is an option of replacement for autologous nerve grafts and can be applied in further clinical studies.

## Additional Information

**How to cite this article:** Muheremu, A. *et al*. Chitosan nerve conduits seeded with autologous bone marrow mononuclear cells for 30 mm goat peroneal nerve defect. *Sci. Rep.* **7**, 44002; doi: 10.1038/srep44002 (2017).

**Publisher's note:** Springer Nature remains neutral with regard to jurisdictional claims in published maps and institutional affiliations.

## Figures and Tables

**Table 1:** Peroneal nerve conduction velocity (m/s) 12 months after the surgery.

Groups	Right side	Left side
BM-MNC	37 ± 5.0	89 ± 8.0
Vehicle	—	90 ± 8.9
Nerve graft	51 ± 6.3	87 ± 7.2

—: No action potential was detected in the vehicle group because the regenerated nerve failed to bridge the long nerve defect.

**Table 2:** Comparison of myelinated axon density, axon diameter and myelin sheath thickness in the regenerated nerve at the distal end among the three groups.

Parameters	BM-MNC group	Vehicle group	Nerve graft group	Normal
Myelinated axon density/mm^2^	18360 ± 2145	—	19220 ± 2378	12400 ± 1830
Axon diameter (μm)	3.67 ± 1.24	—	3.60 ± 0.87	6.12 ± 1.55
Myelin sheath thickness (μm)	0.88 ± 0.11	—	0.97 ± 0.09	1.32 ± 0.10
